# Transcriptional Time Course After Rotator Cuff Tear

**DOI:** 10.3389/fphys.2021.707116

**Published:** 2021-08-06

**Authors:** Laura S. Vasquez-Bolanos, Michael C. Gibbons, Severin Ruoss, Isabella T. Wu, Mario Vargas-Vila, Sydnee A. Hyman, Mary C. Esparza, Donald C. Fithian, John G. Lane, Anshuman Singh, Chanond A. Nasamran, Kathleen M. Fisch, Samuel R. Ward

**Affiliations:** ^1^Department of Bioengineering, University of California, San Diego, San Diego, CA, United States; ^2^Department of Orthopaedic Surgery, University of California, San Diego, San Diego, CA, United States; ^3^Department of Orthopedic Surgery, Kaiser Permanente, San Diego, CA, United States; ^4^Center for Computational Biology and Bioinformatics, Department of Medicine, University of California, San Diego, San Diego, CA, United States; ^5^Department of Radiology, University of California, San Diego, San Diego, CA, United States

**Keywords:** rotator cuff, rotator cuff muscle dysfunction, transcriptome (RNA-seq), time series data analysis, muscle biology, tenotomy, muscle atrophy

## Abstract

Rotator cuff (RC) tears are prevalent in the population above the age of 60. The disease progression leads to muscle atrophy, fibrosis, and fatty infiltration in the chronic state, which is not improved with intervention or surgical repair. This highlights the need to better understand the underlying dysfunction in muscle after RC tendon tear. Contemporary studies aimed at understanding muscle pathobiology after RC tear have considered transcriptional data in mice, rats and sheep models at 2–3 time points (1 to 16 weeks post injury). However, none of these studies observed a transition or resurgence of gene expression after the initial acute time points. In this study, we collected rabbit supraspinatus muscle tissue with high temporal resolution (1, 2, 4, 8, and 16 weeks) post-tenotomy (*n* = 6/group), to determine if unique, time-dependent transcriptional changes occur. RNA sequencing and analyses were performed to identify a transcriptional timeline of RC muscle changes and related morphological sequelae. At 1-week post-tenotomy, the greatest number of differentially expressed genes was observed (1,069 up/873 down) which decreases through 2 (170/133), 4 (86/41), and 8 weeks (16/18), followed by a resurgence and transition of expression at 16 weeks (1,421/293), a behavior which previously has not been captured or reported. Broadly, 1-week post-tenotomy is an acute time point with expected immune system responses, catabolism, and changes in energy metabolism, which continues into 2 weeks with less intensity and greater contribution from mitochondrial effects. Expression shifts at 4 weeks post-tenotomy to fatty acid oxidation, lipolysis, and general upregulation of adipogenesis related genes. The effects of previous weeks’ transcriptional dysfunction present themselves at 8 weeks post-tenotomy with enriched DNA damage binding, aggresome activity, extracellular matrix-receptor changes, and significant expression of genes known to induce apoptosis. At 16 weeks post-tenotomy, there is a range of enriched pathways including extracellular matrix constituent binding, mitophagy, neuronal activity, immune response, and more, highlighting the chaotic nature of this time point and possibility of a chronic classification. Transcriptional activity correlated significantly with histological changes and were enriched for biologically relevant pathways such as lipid metabolism. These data provide platform for understanding the biological mechanisms of chronic muscle degeneration after RC tears.

## Introduction

Rotator cuff (RC) tears are prevalent inthe general population with an incident rate of 20% after the age of60 (Sayampanathan and Andrew, 2017; Collin et al., 2019). Although RC repairs are used to treat this condition, time between tear and repair can influence the success of surgery (Gladstone et al., 2018). However, clinical studies have proven that repair does not improve or reverse the muscle atrophy and fatty infiltration observed at chronic states of disease (Gerber et al., 2000; Gladstone et al., 2018). This highlights the need to better understand what dysfunction in RC tears is leading to persistent muscle atrophy and fatty infiltration to determine potential targets for reversibility.

The rabbit model is an advantageous system to use to study this question due to similar morphological changes such as increased fat, fibrosis, and degeneration (Rubino et al., 2007; Farshad et al., 2012; Valencia et al., 2018; Hyman et al., 2020, 2021, in revision; Vargas-Vila et al., 2021) to what is observed in the supraspinatus (SSP) in humans (Gibbons et al., 2017, 2018b) without the need of a neurectomy as in smaller animal models (Rowshan et al., 2010). The major physiological changes noted in this model include significant decrease in muscle fiber cross-sectional area (CSA) at 4 and 16 weeks, degeneration of muscle fibers with a ∼25% muscle mass reduction after 16 weeks, and an increase in collagen and fat between 4 to 16 weeks (Rubino et al., 2007; Farshad et al., 2012; Valencia et al., 2018; Hyman et al., 2020, 2021, in revision; Vargas-Vila et al., 2021). Understanding the progression of RC disease before repair may help determine the potential effectiveness of a surgical intervention with or without adjuvant therapeutics.

Currently, few human studies have characterized gene expression in RCmuscle after tendon tear and how it relates to the severity of musclechanges (Reardon et al., 2001; Steinbacher et al., 2010; Choo et al., 2014; Chaudhury et al., 2016; Shah et al., 2017; Gibbons et al., 2018a; Ren et al., 2018; Ge et al., 2020; Tashjian et al., 2020), but these data could not be related to a time course neither compared to a healthy control. Gene expression data with two or more time points have been collected in a mouse RC tear model and a rat tenotomy and neurectomy model (Lee et al., 2018; Gumucio et al., 2019; Hu et al., 2019), typically focusing on specific programs and gene overlap over the time series. However, even fewer studies have considered the mechanistic effects of muscle unloading in an animal RC tear model which properly recapitulates the human pathophysiology of fatty infiltration. One example includes the sheep RC tear model, which recapitulates human pathophysiology (Gerber et al., 2004) and has been used to consider the role of mitochondrial dysfunction with transcriptomics at two different time points (2 and 16 weeks post-tenotomy; Flück et al., 2017, 2020), Given the lack of a broad understanding of the progression of RC disease there is an unmet need to investigate the development in a time dependent manner with greater time and transcriptional resolution.

This study aims to establish transcriptional responses to RC tendon tear as a function of time in the rabbit tenotomy injury model. Sequencing for all genes, rather than select ones, allows for an unbiased analysis of transcriptional changes over time. These data may provide a greater understanding of how RC tears progress to a chronic state of decreased muscle quality and function. We hypothesize, based on morphological changes previously observed in this model, that transcription of certain genes is time dependent, where early changes would favor atrophy and inflammation and late changes would favor fatty infiltration, degeneration, and fibrosis.

## Materials and Methods

### Animals

In this study 30 female New Zealand White rabbits (∼6 months, Western Oregon Rabbit Company, Philomath, OR, United States) were used to evaluate post-tenotomy transcriptional changes over time (Vargas-Vila et al., 2021). Females were used due to housing safety concerns regarding mixing gender and the ease of sourcing older female animals. All protocols were approved by the University of California, San Diego Institutional Animal Care and Use Committee (protocol #S11246). All animals were assigned a number ID and cage location upon arrival and then at time of harvest were randomized to one of the study groups. Animals were single housed with food and water ad lib, environmental and food enrichment, and visual access to other animals. There were no adverse events in this study and no animals met the criteria for humane early endpoints.

### Surgical Procedures

Rabbits were anesthetized with a subcutaneous injection of ketamine and xylazine (35 mg/kg ketamine/5 mg/kg xylazine, MWI Veterinary Supply, Boise, ID, United States). Following intubation, 2–4% isoflurane (VetOne, Boise, ID, United States) was utilized to keep the animals under anesthesia for the duration of the surgery. The surgical site was disinfected, and an incision was made through the skin and deltoid muscle overlying the RC. After exposing the SSP tendon, a unilateral tenotomy was performed by transecting the tendon at its footprint on the greater tubercle of the humerus. Surrounding soft tissues were bluntly dissected to permit unobstructed retraction of the tendon. To avoid the formation of tissue adhesions, a Penrose drain (Medline, Northfield, IL, United States) was sutured to the tendon stump. The muscle and skin layers were subsequently sutured and stapled closed, and the animals were allowed to recover. A sham surgery was performed on the contralateral limb of the tenotomy to serve as a control for the procedure where only the skin was cut, tendon isolated, and then stitched up as normal. An E-collar was placed on the animals to prevent suture ripping, and after 10 days, the collar and any remaining staples were removed (Vargas-Vila et al., 2021).

### Muscle Harvesting

After the study, animals were euthanized at 5 time points; 1, 2, 4, 8, and 16 weeks post-tenotomy. At the specified time points, animals were euthanized with an intravenous overdose of pentobarbital (Beuthanasia, 120 mg/kg, MWI Veterinary Supply, Boise, ID, United States). The SSP muscles from both shoulders were harvested and divided into four regions with the central tendon serving as the muscle midline between the anterior and posterior sides of the muscle. These four regions included anterior lateral (A1), posterior lateral (P1), anterior medial (A2), and posterior medial (P2), and one full-muscle thickness fragment was harvested from each location. The harvested muscle regions were pinned to *in vivo* length and flash frozen in liquid nitrogen-chilled isopentane for storage at −80°C (Vargas-Vila et al., 2021).

### RNA Extraction

The muscle samples from the P1 region were removed from −80°C and brought to a cryostat where they were allowed to come up to −20°C. The P1 region was chosen due to consistently presenting the most affected region of muscle in this RC injury model compared to the anterior and medial regions (Vargas-Vila et al., 2021). One notable difference is his region experiences the greatest fiber type changes with increased type II and decreased type I influencing the metabolic activity (Vargas-Vila et al., 2021). A 50–75 mg piece was removed from the center of each pinned region and placed in a pyrogen-free tube. RNA extraction was performed using the QIAGEN Fibrous Tissue mini kit on a QIAGEN Qiacube robot (QIAGEN, Germantown, MD, United States). In brief, the tissue was immersed in buffer RLT and disrupted by bead in the QIAGEN TissueLyser II (QIAGEN, Germantown, MD, United States), before being transferred to the Qiacube for RNA extraction. Samples were digested with Proteinase K, (QIAGEN, Germantown, MD, United States) prior to extraction. A DNase digestion step was included in the protocol. RNA was stored at −80°C.

### RNA Sequencing

Total RNA was assessed for quality using an Agilent Tapestation 4200, and samples with an RNA Integrity Number (RIN) greater than 8.0 were used to generate RNA sequencing libraries using the TruSeq Stranded mRNA Sample Prep Kit (Illumina, San Diego, CA, United States). Samples were processed following manufacturer’s instructions, modifying RNA shear time to 5 min. Resulting libraries were multiplexed and sequenced with 75 basepair (bp) single reads (SR75) to a depth of approximately 25 million reads per sample on an Illumina HiSeq400. Samples were demultiplexed using bcl2fastq Conversion Software (Illumina, San Diego, CA, United States).

### RNAseq Analysis

Quality control of the raw fastq files was performed using thesoftware tool FastQC (Andrews, 2010). Sequencing reads were alignedto the rabbit genome (Ensembl OryCun2.0) using the STAR v2.5.1aaligner (Dobin et al., 2013). Read quantification was performed with RSEM(Li and Dewey, 2011; v1.3.0) and Ensembl annotation (Oryctolagus_cuniculus.OryCun2.0.91.gtf). The R BioConductor packages edgeR (Robinson et al., 2010) and limma (Ritchie et al., 2015) were used to implement limma-voom (Law et al., 2014) followed by empirical Bayes technique for differential expression analysis. Lowly expressed genes were filtered out (cpm > 1 in at least one sample). Trimmed mean of *M*-values (TMM) normalization was applied (Robinson and Oshlack, 2010). The experimental design was modeled upon time point and treatment (∼0 +time_treatment) with contrasts (Tenotomy – Sham) for each time point and all samples. All results will be presented as the tenotomy time point compared to sham unless specified otherwise. From the empirical Bayes result, differentially expressed (DE) genes were defined by an adjusted *P*-value < 0.05 [based on the moderated *t*-statistic using the Benjamini-Hochberg (BH) method to control the false discovery rate (Benjamini et al., 2001)] and a |log2FC| > 1 (Supplementary Data 1). G:Profiler was used to map rabbit Ensemble IDs to human Ensemble IDs, Entrez IDs, and symbols (Raudvere et al., 2019), Supplementary Data 2. Of the 11,535 total genes, 1,210 were not mapped to human and 296 were duplicates and were removed for the analysis. The resulting genes with Entrez IDs correspond to the set of “background or detected genes” consisting of 10,029 genes. We removed one 8 week sample that we identified as an outlier using PCA plots and unsupervised hierarchical clustering. With *n* = 6 animals per group, we were sufficiently powered to identify significantly DE genes with a power of 72%. Volcano plots were created using Enhanced Volcano package (v.1.60). Heatmaps were created using clustermap in the seasborn package (Waskom, 2021). The Venn Diagram was produced using Van de Peer lab tools (Van de Peer Lab, 2021). RT-qPCR validation was not used in this study due to the robust nature of RNAseq methods and data analysis and supporting literature (Feng et al., 2010; Coenye, 2021).

### Enrichment Analysis

Assignment of functional categories was based on the Gene Ontology(GO) categories “Biological process,” “Molecular function,” and“Cellular component.” Enrichment analysis of GO categories wasperformed in R (version 4.0.2; http://www.r-project.org) using the “weight01” method from the Bioconductor topGO (v. 2.40.0) package with the org.Hs.eg.db_3.11.4 human database (Carlson, 2019; Alexa and Rahnenfuhrer, 2020). Node size was set to 10, and Fisher’s exact test was used for assessing GO term significance. Overrepresentation of functional categories was calculated for DE genes as compared with the 10,030 “background” genes, and significant GO terms were identified as those having *P*-value < 0.05 (Supplementary Data 3). KEGG analysis was also done in R using KEGGREST package (v. 1.28.0) with list of pathways and genes. A Wilcox rank-sum test was performed for each pathway, the Entrez ID along with the adjusted *p*-values of the gene expression as input. Testing whether *p*-values of genes included in that pathway are smaller than outside *p*-values. Overrepresentation of KEGG pathways was calculated for DE genes as compared with the 10,030 “background” genes, and significant KEGG pathways were identified as those having a *p*-value < 0.05 (Supplementary Data 4).

### Gene Pathways of Interest

To highlight the changes in common genetic programs of interest, theRC muscle literature was searched to build a list of essential genes(Supplementary Data 5) found in programs such as myogenesis, inflammation, adipogenesis, and fibrosis (Gibbons et al., 2018a). Additional genes relating to adipogenesis identified as correlated with RC radiographic assessments (Shah et al., 2017) were also included. The final lists were compared to other RC transcriptome analyses in other animal models (Lee et al., 2018; Gumucio et al., 2019; Flück et al., 2020) for confirmation of overlap although in more broadly described categories.

### Correlation Analysis

**FIGURE 1:**
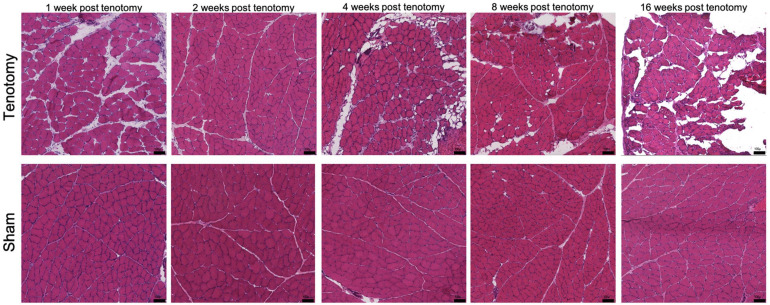
Representative histological H&E sections of muscle at each time point for post-tenotomy and sham. Demonstrating over time a decrease in muscle CSA, muscle mass, increase in collagen content and fat reported in Vargas-Vila et al. (2021). Scale bar 100 μm.

Weighted correlation network analysis (WGCNA) was performed usingWGCNA R package (v. 1.70-3; Langfelder and Horvath, 2008) withtranscriptional and phenotypic data (Supplementary Data 6) with the tenotomy only samples as there was no significance found in the combined analysis. This analysis works by building an unbiased network of modules which represents a cluster of genes, and then correlations (Supplementary Data 7) can be investigated with phenotype traits through gene membership. The phenotypic data included in this study is fiber area, central nucleation, fat quantification, collagen content, and degeneration, all of which are reported in detail elsewhere (Vargas-Vila et al., 2021) but are from the same animals used in this study. However, Hematoxylin and Eosin-stained (H&E) stained sections of representative muscles in each group, at each time point, can be seen in Figure 1.

## Results

### Transcriptome Profiles Changed From Acute to Chronic State

Visually there were obvious structural muscle changes over time between post-tenotomy and sham samples (Figure 1). Changes included decreased muscle CSA, increased muscle degeneration, and increased fat and collagen deposition (Vargas-Vila et al., 2021).

**FIGURE 2:**
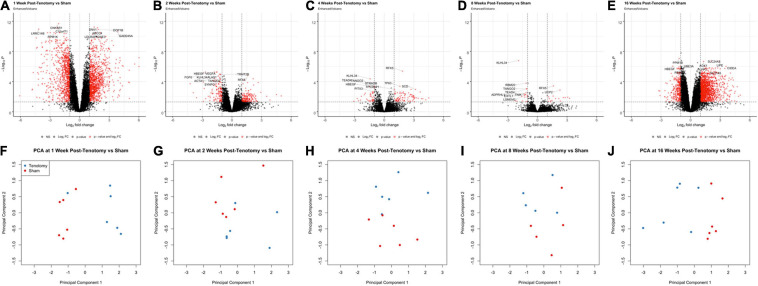
Volcano plots **(A–E)** highlight differentially expressed (DE) genes (red dots) at each time point post-tenotomy defined as a logFC > 1 and an adjusted *p*-value < 0.05. The genes labeled in each volcano plot **(A–E)** consist of the top 10 genes with the smallest *p*-value. Corresponding PCA plots **(F–J)** at each time point demonstrate that the analytical methods separate tenotomy and sham.

At 1 week post-tenotomy, the initial acute time point, there was substantial number of significantly up- and down-regulated DE genes (Figure 2A), followed by a sharp decrease in differentially regulated transcripts at 2 (Figure 2B), 4 (Figure 2C), and 8 weeks post-tenotomy (Figure 2D). However, at 16 weeks post-tenotomy (Figure 2E) there was a resurgence in the number of DE genes, which considers this time point at a chronic state because although the expression is similar in intensity to 1 week post-tenotomy (Figure 2A), it had more up-regulated genes than down-regulated genes. The difference between the labeled genes in Figures 2A–E include that the genes at 1 week post-tenotomy do not appear in the described top 10 genes at any other time point, meanwhile there was overlap with the other time points, although with no clear commonality. Each time point’s corresponding principal component analysis (Figures 2F–J) illustrated that standard analytical methods easily separate tenotomy from sham.

**FIGURE 3:**
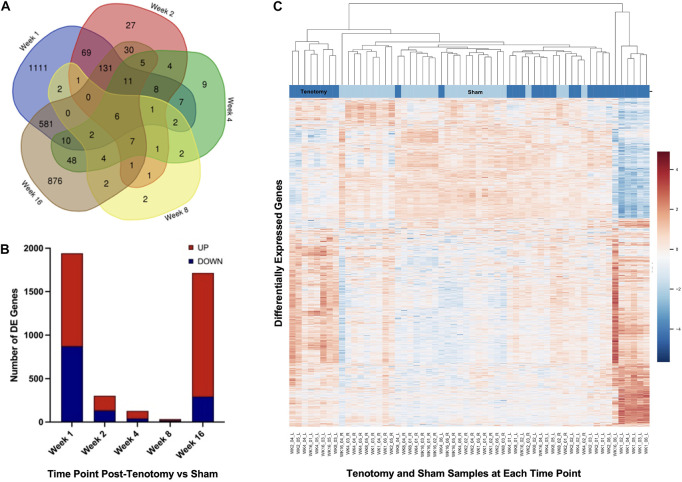
Distribution of samples by tenotomy vs sham and DE genes over each time point post-tenotomy. Venn diagram **(A)** highlights the DE genes at each time point and the overlap with other time points. The bar chart **(B)** displays the number of DE genes which are up or down regulated at each timepoint. Data in the heatmap **(C)** is presented as normalized expression for each tenotomy and sham sample at each time point post-tenotomy with a *z*-score scale by rows and an average hierarchical clustering by columns.

The Venn diagram (Figure 3A) shows the distribution of DE genes between each time point post-tenotomy, where 1 week had 1,111 unique genes, 2 weeks with 27, 4 weeks with 9, 8 weeks with 2, and 16 weeks post-tenotomy with 876. There are 6 DE genes [KLHL34, LSMEM2 (Leucine Rich Single-Pass Membrane Protein 2), UCP2 (Uncoupling Protein 2), TP63 (Tumor Protein P63), MYT1L (Myelin Transcription Factor 1 Like), ADPRHL1 (ADP-Ribosylhydrolase Like 1)] in common at all the time points which generally have to do with energy or transcription factors. The 1 week and 16 weeks post-tenotomy time points uniquely shared 581 DE genes (only 25% of their respective total DE genes). The total amount of DE genes by week was 1,942 with 1,069 up and 873 down for 1 week, 303 with 170 up and 133 down for 2 weeks, 127 with 86 up and 41 down for 4 weeks, 34 with 16 up and 18 down for 8 weeks and 1,714 with 1,421 up and 293 down for 16 weeks post-tenotomy (Figure 3B). Despite 1 and 16 weeks post-tenotomy shared an approximately equivalent number of DE genes, instead of the DE genes being split by up- and down-regulated as seen at the 1 week post-tenotomy, the 16 weeks post-tenotomy demonstrated 83% of up-regulated DE genes (Figure 3B). This contrast between 1 and 16 weeks post-tenotomy can also be demonstrated by the clustering in the heatmap. The clustering grouped 5 out of 6 of 1 week post-tenotomy samples separately from 16 weeks post-tenotomy (Figure 3C). The sham samples generally clustered together with some tenotomy sample exceptions (Figure 3C).

### Enrichment Analysis

**FIGURE 4:**
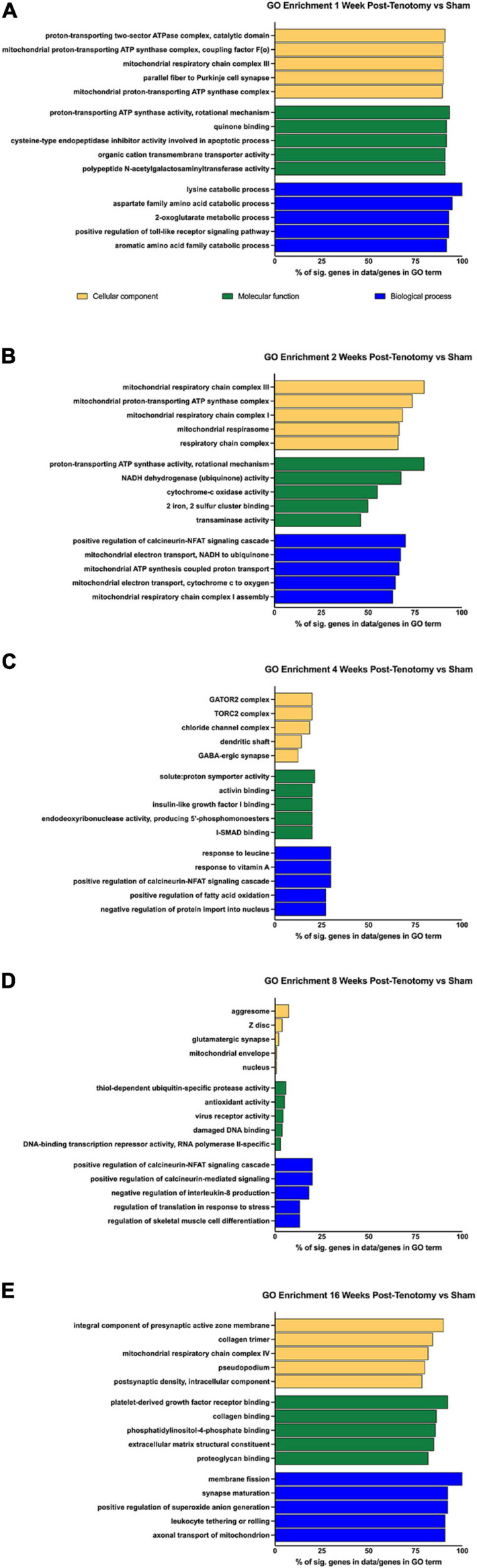
GO enrichment analysis for **(A)** 1 week post-tenotomy, **(B)** 2 weeks post-tenotomy, **(C)** 4 weeks post-tenotomy, **(D)** 8 weeks post-tenotomy, and **(E)** 16 weeks post-tenotomy. Data presented as top 5 greatest gene coverage (# of significant genes/total genes in GO term) in each category: cellular component (yellow), molecular function (green), and biological process (blue).

To determine the larger scale function the DE genes, overrepresentation analyses were performed using GO and KEGG at each time point (Figure 4). For 1 week post-tenotomy, the GO cellular component (yellow bars – Figure 4A) was enriched for transcripts relating to mitochondria. The molecular function (green bars – Figure 4A) was enriched for transcripts related to energy, and transporter and transferase activity. Biological processes (blue bars – Figure 4A) were enriched regarding catabolic, metabolic, and signaling specific pathways.

Gene ontology analysis at 2 weeks post-tenotomy continued to be enriched for components relating to mitochondria, molecular function relating to enzymes of the electron transport chain for ATP, NADH, cytochrome-c, and iron-sulfur binding (Figure 4B). The electron transport chain activity continued to be in enriched in the biological process along with positive regulation of the calcineurin-NFAT signaling cascade (Figure 4B).

At 4 weeks post-tenotomy, the cellular components were enriched for complexes related to signaling and neurotransmission (Figure 4C). Molecular function at 4 weeks was enriched for binding (activin, IGF1, and I-SMAD), catalytic activity on DNA, and proton transport (Figure 4C). There was continued enrichment of positive regulation of the calcineurin-NFAT signaling cascade at 4 weeks in biological process along with response of vitamin A and leucine (Figure 4C). This was the first time point where positive regulation of fatty acid oxidation appeared along with negative regulation of protein import into the nucleus.

The percent of genes in a given GO term decreases over time and was at its lowest at 8 weeks post-tenotomy, with most having 25% or less genes in the term (Figure 4D). The cellular components had broad enrichment at 8 weeks, ranging from aggresome, *Z*-disk, synapse, mitochondrial envelope to nucleus (Figure 4D). Interestingly, damaged DNA binding and transcription repressor activity appeared enriched in molecular function at 8 weeks, along with ubiquitin protease activity, virus receptor activity, and antioxidant activity (Figure 4D). Positive regulation of calcineurin-related signaling again was enriched in biological process at 8 weeks with more regulation related to IL-8, translation in response to stress, and skeletal muscle cell differentiation.

An increase of ∼50% in significant genes within a GO term from 8 weeks post-tenotomy to 16 weeks post-tenotomy was seen with a range of cellular components enriched for the synapse, collagen trimer, mitochondrial respiratory chain complex I, and pseudopodium (Figure 4E). Molecular function at 16 weeks was enriched for binding (PDGF, collagen, proteoglycan, and phosphatidylinositol-4-phosphate) and extracellular matrix constituents (Figure 4E). Positive regulation of reactive oxygen species (ROS) generation was enriched in biological process at 16 weeks, along with synapse maturation, axonal transport of mitochondrion, leukocytes rolling, and membrane fission (Figure 4E).

**FIGURE 5:**
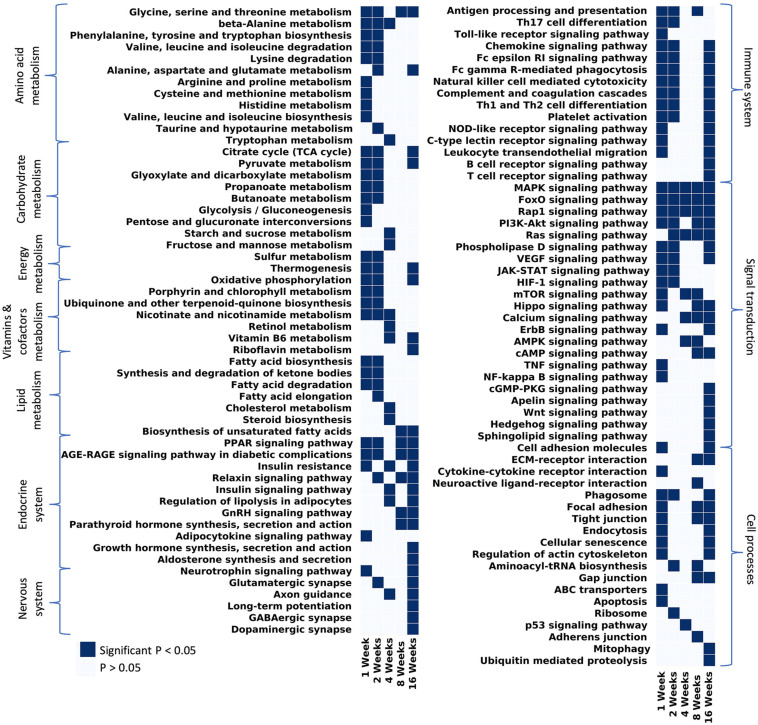
Considering all pathways that have at least 1 significant *p*-value and filtering out disease/tissue specific pathways, the remaining pathways were grouped by KEGG hierarchy into amino acid metabolism, carbohydrate metabolism, vitamins and cofactors metabolism, energy metabolism, lipid metabolism, endocrine system, nervous system, immune system, signal transduction, and cell processes.

KEGG analyses highlighted changes of broader pathways over time (Figure 5). At 1 week post-tenotomy, all metabolism terms were significantly enriched and generally continued into 2 weeks post-tenotomy (Figure 5 – metabolism). Metabolism at 4 weeks post-tenotomy identified new enriched metabolism terms such as tryptophan metabolism (Figure 5 – amino acid metabolism), retinol metabolism (Figure 5 – vitamins and cofactors metabolism), sugar metabolism (Figure 5 – carbohydrate metabolism), cholesterol metabolism, and steroid biosynthesis (Figure 5 – lipid metabolism). Only glycine, serine, threonine metabolism (Figure 5 – amino acid metabolism), and biosynthesis of unsaturated fatty acid (Figure 5 – lipid metabolism) were enriched at 8 weeks post-tenotomy. By 16 weeks post-tenotomy there were only a few pathways in each metabolism category that were enriched significantly. In the signaling transduction cluster, MAPK, FoxO, Rap1 signaling pathways were the only terms enriched at all time points. Most terms in this cluster were enriched at 16 weeks post-tenotomy with 5 pathways (cGMP-PKG, Apelin, Wnt, Hedgehog, and Sphingolipid signaling pathway) that became enriched only at this time point. Focusing on the immune system cluster, there was a clear activation of all terms at 1 week which decreased slightly by 2 weeks, was not present at 4 weeks and only antigen processing and presentation was enriched at 8 weeks, but by 16 weeks post-tenotomy there was a reactivation of many of the 1 week terms in addition to B and T cell receptor signaling pathway (Figure 5 – immune system). There were a few enriched terms in the endocrine system cluster at 1, 2, and 4 weeks with double the number at 8 weeks and over 9 at 16 weeks post-tenotomy (Figure 5 – endocrine system). At 4 weeks post-tenotomy, regulation of lipolysis in adipocytes, insulin resistance and signaling pathway were enriched and continued to be at 16 weeks post-tenotomy along with many more endocrine system terms. 16 weeks post-tenotomy also had many significantly enriched terms in the nervous system cluster which contrasted with the behavior at 1 week post-tenotomy. The cell processes cluster highlighted terms with the cell environment where 1 week post-tenotomy was enriched for apoptosis, ABC transporters, cytokine-cytokine receptor interaction, 2 weeks with ribosome, 4 weeks with p53 signaling pathway, 8 weeks with neuroactive ligand-receptor interaction, adherens junction, and 16 weeks with mitophagy and ubiquitin mediated proteolysis.

### Gene Programs of Interest Changed Over Time After Tear

**FIGURE 6:**
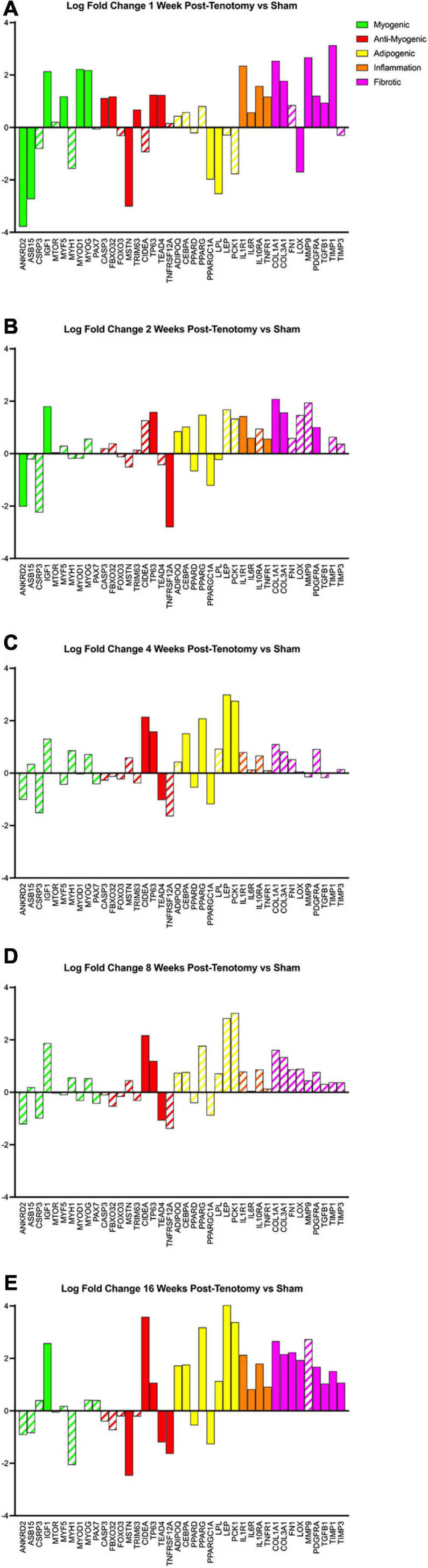
Changes in transcriptome of genetic programs of interest at **(A)** 1 week post-tenotomy, **(B)** 2 weeks post-tenotomy, **(C)** 4 weeks post-tenotomy, **(D)** 8 weeks post-tenotomy, and **(E)** 16 weeks post-tenotomy. Log fold change (logFC) is the difference of tenotomy and sham. Solid bars represent a significant adjusted *p*-value (*p* < 0.05) and partially filled in bars are not significant. Green bars represent myogenic related genes, red represents anti-myogenic, yellow represents adiopogenic, orange represents inflammation, and pink represents fibrotic genes. Data are presented as average logFC.

Using a literature-driven approach, specific genes from the literature relating to myogenic, anti-myogenic (suppressing muscle formation, cell death, and degradation), adipogenic, inflammation, and fibrotic programs were highlighted across the five time points (Figure 6). The myogenic program (Figure 6 – green bars) was activated the strongest after 1 week post-tenotomy (Figure 6A) and was faint or not significant at all other time points (Figures 6B–E). The anti-myogenic program (Figure 6 – red bars) was also expressed strongly at 1 week post-tenotomy with continued expression at all the time points, although with fewer genes at the 2–8 week time points, and then presented a second robust response at 16 weeks. Although some genes within the adipogenic program (Figure 6 – yellow bars) were significantly expressed in 1 and 2 weeks, the entire program was fully expressed initially at 4 weeks and again at 16 weeks post-tenotomy. Inflammation (Figure 6 – orange bars) was observed with a large expression at 1 week post-tenotomy and decreasing through 8 weeks, with a resurgence at 16 weeks. The fibrotic program (Figure 6 – pink bars) was also initially expressed at 1 week with fewer expressed genes at 2 weeks followed by no significant genes at 4 and 8 weeks (Figures 6C,D – dashed bars) and another robust response at 16 weeks post-tenotomy (Figure 6E).

### Transcriptional Data Correlations With Phenotypic Traits

Weighted correlation network analysis revealed gene modules significantly related to phenotype characteristics quantified by histology (Supplementary Figure 1). Modules 10 and 4 encompass the greatest number of significant correlations with phenotype traits, and genes in these modules were significantly enriched for interesting pathways related to lipid metabolism, and general RNA activity (Supplementary Data 8). The phenotype traits degeneration (*p* = 0.07) and collagen content (*p* = 0.03) were only correlated with module 10, which is enriched for lipid metabolism. The strongest correlation coefficient (-0.71, *p* = 2e-05) is associated with the centralized nuclei trait (a marker of muscle degeneration-regeneration) and module 13 which is enriched for pathways related to DNA binding and regulation. Centralized nuclei trait is also correlated significantly to modules 9, 11, and 14 and these genes are enriched for skeletal muscle adaptation/fast-slow fiber transition, endoplasmic reticulum related protein binding, and ubiquitin-like protease activity, respectively, (Supplementary Figure 1 and Supplementary Data 8). Module 7 is significantly associated with interfascicular fat and fat and degeneration phenotype traits whose genes are enriched for interesting pathways as Ragulator complex which is anchored to lipid rafts in late endosomes and protein import related to peroxisomes membrane (Supplementary Figure 1 and Supplementary Data 8).

## Discussion

The purpose of this study was to establish transcriptional changes as a function of time in a rabbit RC tear model. We hypothesize, based on morphological changes previously observed in this model, that transcription of certain genes is time dependent, where early changes would favor atrophy and inflammation and late changes would favor fatty infiltration, degeneration, and fibrosis. There were clear transcriptional differences between each time point, which support the concept of differential timing of inflammation, adipogenesis, fibrosis, and cell death programs that lead to muscle atrophy, degeneration, and fatty infiltration. Likewise, phenotypic traits correlated significantly with gene groupings in unbiasedly defined modules which were enriched for biological relevant pathways such as lipid metabolism.

At 1 week post-tenotomy, as expected, there was a large transcriptional response in both up- and down regulation (Figures 2A, 3B). Functional enrichment elucidates that these genes are related to the immune system, energy metabolism, catabolism and a wide range of signaling pathways we would expect to see at an acute response to injury (Figures 4A, 5A). 2 weeks post-tenotomy the response trends in areas of metabolism, particularly with the mitochondria (Figure 4B), signaling pathways, and immune system with fewer enriched terms (Figure 5). Overall, 2 weeks post-tenotomy had a more muted response than the previous week with greater emphasis on ubiquinone, a metabolite involved in the electron transport chain in the mitochondria and free-radical scavenger antioxidant, related build up (Figure 4B).

A turning point appeared to occur at 4 weeks post-tenotomy where the expression, despite decreasing from 2 weeks (Figure 2C), shares a greater overlap of DE genes with the 16 weeks time point as opposed to 1 and 2 weeks (Figure 3A). The enrichment analyses supported the gene shift toward lipids and adipogenesis, with enriched pathways of positive regulation of fatty acid oxidation (Figure 4C) and regulation of lipolysis in adipocytes (Figure 6). Likewise, when literature-specific adipogenesis genes were considered, this time point demonstrated significant upregulation of these genes (Figure 6C).

The 8 weeks post-tenotomy time point, with only 34 DE genes, was the time point with the lowest levels of DE expression demonstrating a possible trend toward steady state of transcription expression (Figures 2D, 3). These genes are related to GO terms associated with DNA damage binding, transcription repression, regulation of translation in response to stress, and interestingly aggresome, which serves as a location for misfolded proteins and is used when there is too much protein degradation for the cell to handle (Figure 4D). These changes are all associated with low transcriptional activity and degradation. ECM-receptor interaction, including neuroactive ligand-receptor interaction, GAP junctions first appeared enriched at this time point highlighting a change in the cell environment (Figure 6). Given how few significant genes there were at 8 weeks post-tenotomy, only CIDEA, TP63, and TEAD4 from the literature specific genes (Figure 6D) were significantly expressed in the anti-myogenic category, which interestingly, are related to apoptosis.

At 16 weeks post-tenotomy, a resurgence in expression, well after the acute stage response, suggests the possibility of a unique, chronic transcriptional signature. Based on morphology from previous studies (Vargas-Vila et al., 2021), this was when fatty infiltration was most present. Transcriptionally, there are 876 unique DE genes at 16 weeks post-tenotomy not present at any other time point highlighting the difference in response from 1 week post-tenotomy (Figure 2A). Positive regulation of superoxide (a type of ROS) anion generation, ubiquitin mediated proteolysis, general immune system response was enriched at this time point (Figure 4E). Similarly, extracellular matrix (ECM) binding such as collagen, proteoglycans, neuronal activity relating to synapse components, maturation and axonal transport of mitochondrion are enriched (Figure 4E). Mitophagy, in particular, was enriched at this time point suggesting there was sufficient mitochondrial damage/stress that needed to be degraded by autophagy. Not including the myogenic specific genes, all literature-based programs are significantly expressed and more so than at any other time point (Figure 6E).

The distribution of DE genes with the first acute time point having the most and the decrease of expression (Figure 2) was similar to other transcriptional time-series studies (Lee et al., 2018; Gumucio et al., 2019) in a mouse RC tear model (1 and 4 weeks) and a rat (10, 30, and 60 days) RC tear and denervation model. However, neither of these studies captured what was observed at the 16 weeks post-tenotomy time point (Figure 2E), which was a resurgence of expression to a similar level of the most acute time point at 1 week post-tenotomy, but with 876 new genes (Figure 3A) and a greater ratio of up-regulated genes instead of down-regulated (Figure 3B) where autophagy and ECM binding dominate. These studies also demonstrated a large overlap of DE genes at all recorded time points (Lee et al., 2018; Gumucio et al., 2019; Hu et al., 2019), which does not encompass the RC tear progression over time since this study only recorded 6 genes in common at all time points (Figure 3). This is an advantage of this study due to the time and sequence resolution used in order to capture unique time points during RC tear progression. In regard to enrichment there were similar trends in expression of ECM related genes increasing over-time in a rat tenotomy and neurectomy model (Gumucio et al., 2019) which was similar to the genes in the literature-defined fibrotic program that increased at 16 weeks post-tenotomy in this study (Figure 6).

In a more clinically relevant sheep RC tear model (Flück et al., 2017, 2020), with two time points (2 and 16 weeks) highlighted that there were 350 transcripts reported different at either time point. Enrichment analyses highlighted similarities with pathways related to focal adhesions, calcium binding, and extracellular space, which were also enriched at 16 weeks post-tenotomy in our study (Figure 5). Comparing to a human qPCR study of torn RC muscle (Gibbons et al., 2018a), the gene expression across the cell programs in our study at 16 weeks post-tenotomy (Figure 6E) closely resembled biopsies taken from high-fat characterized muscle compared to no-muscle or intact biopsies (Gibbons et al., 2018a). Highlighting essential genes from the literature, we observed the adipogenic pathway expressed starting at 4 and 16 weeks post-tenotomy (Figure 6). Likewise, the difference between 1- and 16 weeks post-tenotomy emphasizes how expression shifts toward more adipogenic and fibrotic genes.

Given a unique, chronic transcriptional profile which emphasized ROS build up, protein degradation, change in ECM/cell environment, the cause(s) leading to these changes merit further investigation to better understand the mechanisms of chronic fatty, fibrotic muscle atrophy observed in RC disease. Some intriguing possibilities based on these data are; changes in metabolism with the mitochondria and oxidative stress, late stage dysregulation of the inflammatory system, or a combination of metabolic and inflammatory dysregulation encouraging autophagy. These potential hypotheses, and other based on these unique data, should be tested mechanistically by deliberately manipulating this now better-defined system.

We are unaware of any other correlation between RNAseq data and muscle structural changes over time in RC muscle. Specifically, unbiased gene sets were correlated with histological measurements in our tenotomy samples, where certain gene sets correlated positively with most histological measurements (Supplementary Figure 1). The fact that there was an obvious statistical correlation between transcriptional activity and structure reinforces our confidence in the physiological significance of the RNAseq data presented in this study. Importantly, although sets of genes, as opposed to individual genes or even pathways, are likely most relevant to the broad degenerative changes seen in tenotomized muscle, we are not implying that direct cause-effect relationships between specific gene set dysregulation and structural changes as observed in this analysis. That being said, the connection between altered lipid metabolism, DNA regulation, and ubiquitination are all logically appealing pathways to probe contributing to future studies in the muscle physiology field along with better understanding RC tear injury dysfunction. Future studies will need to manipulate gene sets, or pathways, positively and negatively to determine if they are mechanistically related to degeneration. Additionally, given the heterogeneity of muscle degeneration across individual muscles, relating transcriptional activity to specific regions of muscle will be a valuable technological advance.

## Conclusion

Defining transcriptional changes in a RC tear model such as rabbit, which is more similar to human RC disease progression, allows for the possibility of further mechanistic studies to understand the muscle dysfunction leading to muscle atrophy and fatty infiltration observed. The field needs to better understand the progression of tear in order to make more informed decisions regarding RC repair and therapeutics. This study outlines the timeline of transcriptional changes in muscle after RC tear such as the immune response at 1 week post-tenotomy, mitochondrial activity at 2 weeks post tenotomy, adipogenesis active at 4 weeks post-tenotomy, transcriptional steady state at 8 weeks post-tenotomy, and not previously reported resurgence of transcription at 16 weeks post-tenotomy, which may represent a chronic transcriptional signature, and correlates gene sets to structural muscle changes over time.

## Data Availability Statement

The data discussed in this publication have been deposited in NCBI’sGene Expression Omnibus (Edgar et al., 2002) and are accessible through GEO Series accession number GSE173234 (https://www.ncbi.nlm.nih.gov/geo/query/acc.cgi?acc=GSE173234).

## Ethics Statement

The animal study was reviewed and approved by University of California, San Diego Institutional Animal Care and Use Committee.

## Author Contributions

MG, MV-V, JL, AS, and SW were responsible for the conceptionand design of the study. LV-B, MG, SR, MV-V, IW, SH, ME, DF, JL, AS, CN, and KF contributed to collection andanalysis of data. LV-B was responsible for the design and draftingof the manuscript. All authors revised the manuscript andgave final approval.

## Conflict of Interest

The authors declare that the research was conducted in the absence of any commercial or financial relationships that could be construed as a potential conflict of interest.

## Publisher’s Note

All claims expressed in this article are solely those of the authors and do not necessarily represent those of their affiliated organizations, or those of the publisher, the editors and the reviewers. Any product that may be evaluated in this article, or claim that may be made by its manufacturer, is not guaranteed or endorsed by the publisher.
