# Corrigendum: Transcriptional Time Course After Rotator Cuff Tear

**DOI:** 10.3389/fphys.2021.775297

**Published:** 2021-10-29

**Authors:** Laura S. Vasquez-Bolanos, Michael C. Gibbons, Severin Ruoss, Isabella T. Wu, Mario Vargas-Vila, Sydnee A. Hyman, Mary C. Esparza, Donald C. Fithian, John G. Lane, Anshuman Singh, Chanond A. Nasamran, Kathleen M. Fisch, Samuel R. Ward

**Affiliations:** ^1^Department of Bioengineering, University of California, San Diego, San Diego, CA, United States; ^2^Department of Orthopaedic Surgery, University of California, San Diego, San Diego, CA, United States; ^3^Department of Orthopedic Surgery, Kaiser Permanente, San Diego, CA, United States; ^4^Center for Computational Biology and Bioinformatics, Department of Medicine, University of California, San Diego, San Diego, CA, United States; ^5^Department of Radiology, University of California, San Diego, San Diego, CA, United States

**Keywords:** rotator cuff, rotator cuff muscle dysfunction, transcriptome (RNA-seq), time series data analysis, muscle biology, tenotomy, muscle atrophy

In the original article, there was a mistake in Figure 3 as published. **An older version of the figure was published by mistake**. The corrected Figure 3 appears below.

**Figure 3:**
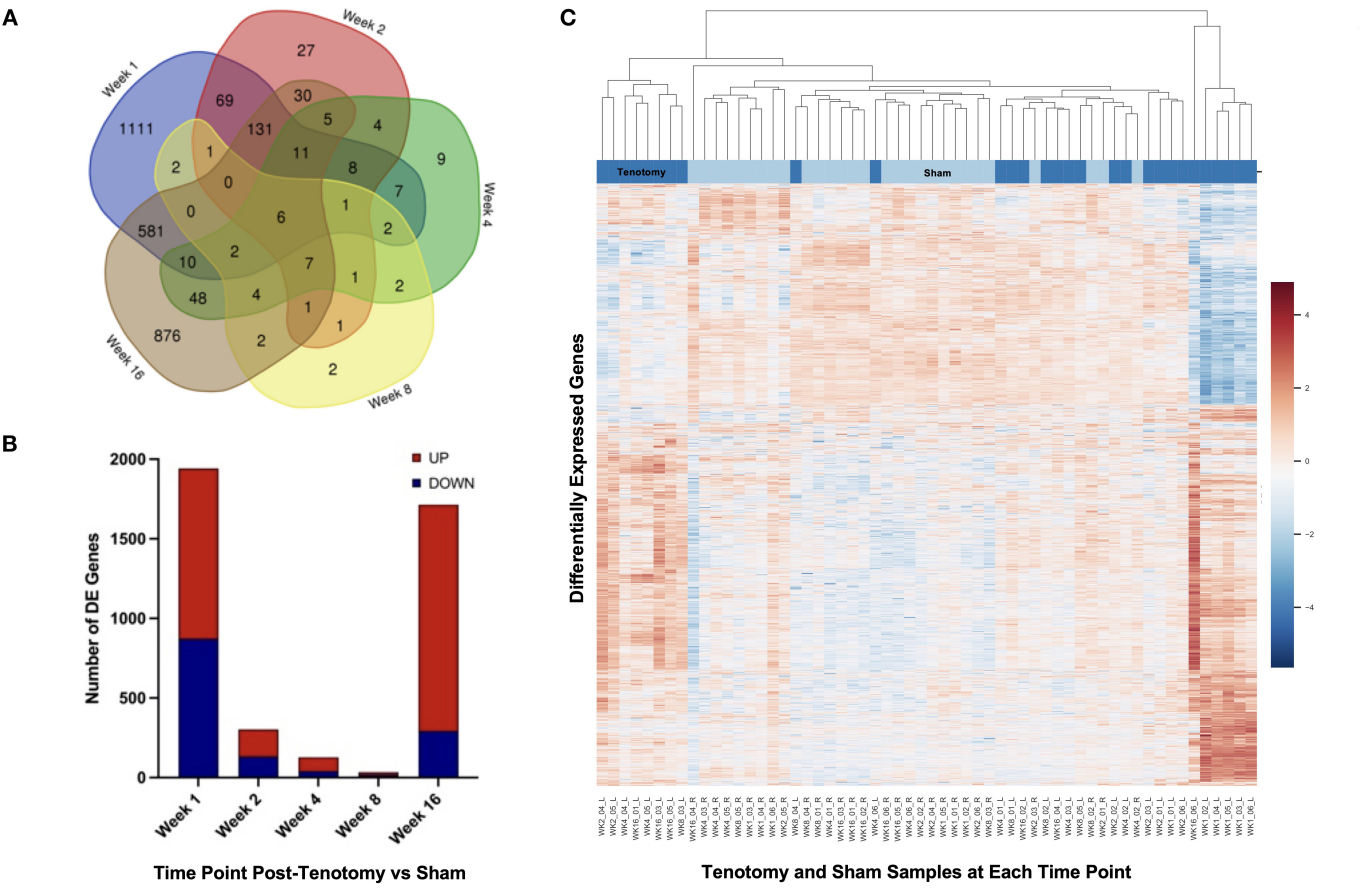
Distribution of samples by tenotomy vs sham and DE genes over each time point post-tenotomy. Venn diagram **(A)** highlights the DE genes at each time point and the overlap with other time points. The bar chart **(B)** displays the number of DE genes which are up or down regulated at each timepoint. Data in the heatmap **(C)** is presented as normalized expression for each tenotomy and sham sample at each time point post-tenotomy with a z-score scale by rows and an average hierarchical clustering by columns.

The authors apologize for this error and state that this does not change the scientific conclusions of the article in any way. The original article has been updated.

## Publisher's Note

All claims expressed in this article are solely those of the authors and do not necessarily represent those of their affiliated organizations, or those of the publisher, the editors and the reviewers. Any product that may be evaluated in this article, or claim that may be made by its manufacturer, is not guaranteed or endorsed by the publisher.

