# Supplementary material for: Transcriptional Time Course After Rotator Cuff Tear
Source: Front Physiol. 2021 Aug 6;12:707116. doi: 10.3389/fphys.2021.707116 (PMC8378535; doi:10.3389/fphys.2021.707116)
Supplement: Supplementary file 9 [file Data_Sheet_9.PDF]

Supplementary Material

1 Supplementary Data

Excel 1. DE matrix where a 1 represents an up-regulated differentially expressed gene and a -1 represents a down-regulated differentially expressed gene. The DE genes for each time point are included.

Excel 2. Toptable includes the mapping from rabbit Ensembl to human Ensembl “GENEID”, ENTREZID, and SYMBOL for all genes recorded. As well as, for each time point and each gene, it includes the logFC, AveExpr, t, p-value, adjusted p-value, and B.

Excel 3. GO terms for biological process (BP), molecular function (MF) and cellular component (CC) for each time point includes GO ID, name of term, number of genes in term, number significant, number of genes expected, p-value, and percent of significant genes over all genes in term. Yellow highlight represents the terms included in Figure 3 in the paper, and a space included in each group separates significant ($p<0.05$) from not significant.

Excel 4. All KEGG results includes the pathway code, name, p-value, number of genes annotated in pathway, and total genes related to pathway. Then all significant pathways ($p<0.05$) for at least one time point were separated and then filtered to remove disease and less relevant terms. Final sheet includes the final list and grouping used to create heatmap in Figure 4.

Excel 5. List of literature based genes and their categories from Figure 5.

Excel 6. Metadata of tenotomy samples and phenotypic characteristics collected used for WGCNA.

Excel 7. Full correlation matrix of module number by phenotypic trait, including correlation values (in the first 8 columns) and p-values (in the following 8 columns).

Excel 8. GO enrichment analysis of genes assigned to each module with module size, number of genes found in GO terms as background, the rank of the enrichment (top 10), p-value, number of module genes and background, GO term ID, ontology, name and definition.

2 Supplementary Figures

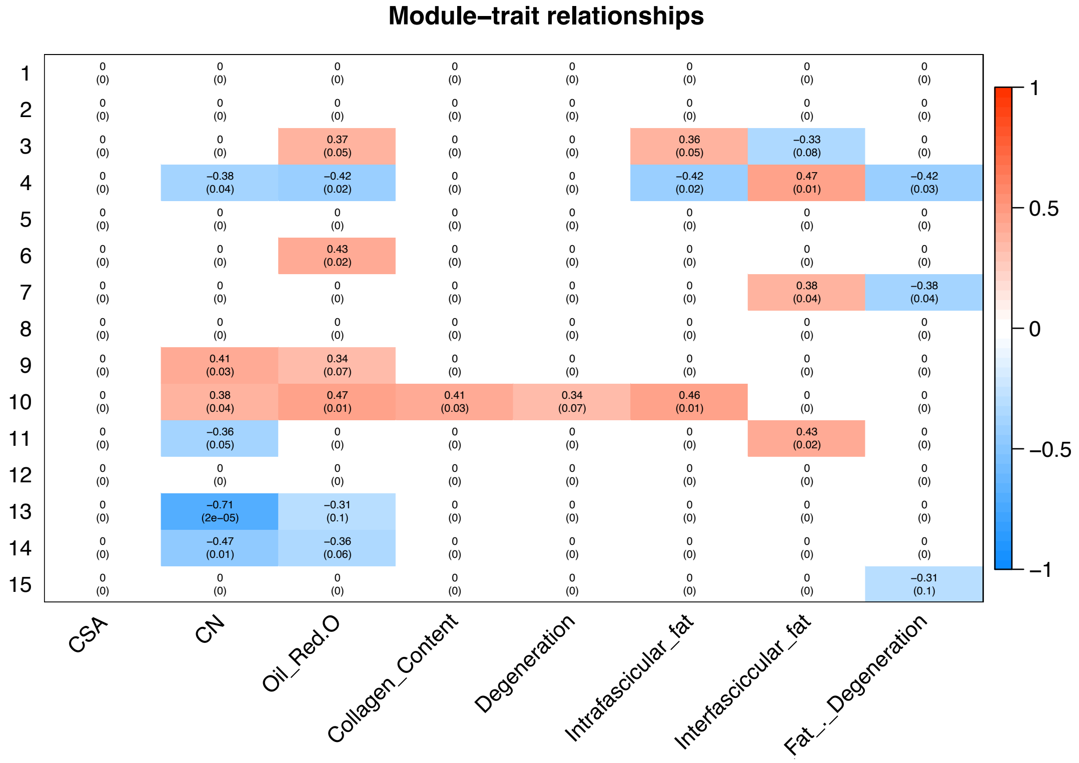

Supplementary Figure 1. The modules are listed 1-15 on the left and the phenotypic traits are on the bottom. The scale bar represents the correlation coefficient and is the first number listed in each cell, and the number in parentheses is the p-value. Only cells with a p-value equal or less than 0.1 were selected to be displayed and the white cells with zeros represent cells with a p-value > 0.1 for clarity of which modules correlate with which phenotypic traits.
